# Hyper-IgE and Carcinoma in CADINS Disease

**DOI:** 10.3389/fimmu.2022.878989

**Published:** 2022-05-16

**Authors:** Leonora Pietzsch, Julia Körholz, Felix Boschann, Mildred Sergon, Batsukh Dorjbal, Debra Yee, Vanessa Gilly, Eva Kämmerer, Diana Paul, Clemens Kastl, Martin W. Laass, Reinhard Berner, Eva Maria Jacobsen, Joachim Roesler, Daniela Aust, Min A. Lee-Kirsch, Andrew L. Snow, Catharina Schuetz

**Affiliations:** ^1^ Department of Pediatrics, University Hospital Carl Gustav Carus, Technische Universität Dresden, Dresden, Germany; ^2^ Institute of Medical Genetics and Human Genetics, Charité - Universitätsmedizin Berlin, Corporate Member of Freie Universität Berlin, Berlin Institute of Health, Humboldt-Universität zu Berlin, Berlin, Germany; ^3^ Department of Pathology, University Hospital Carl Gustav Carus, Technische Universität Dresden, Dresden, Germany; ^4^ Department of Pharmacology and Molecular Therapeutics, Uniformed Services University of the Health Sciences, Bethesda, MD, United States; ^5^ Hautarztpraxis Freiberg, Freiberg, Germany; ^6^ Universitäts Centrum für Seltene Erkrankungen, University Hospital Carl-Gustav-Carus, Technische Universität Dresden, Dresden, Germany; ^7^ Department of Pediatrics, University Medical Center Ulm, Ulm, Germany; ^8^ Core Unit for Molecular Tumor Diagnostics (CMTD), National Center for Tumor Diseases (NCT) Dresden, Dresden, Germany; ^9^ Nationales Centrum für Tumorerkrankungen (NCT)/Universitäts KrebsCentrum (UCC) Biobank Dresden, National Center for Tumor Diseases (NCT) Dresden and University Hospital Carl Gustav Carus, Technische Universität Dresden, Dresden, Germany

**Keywords:** CARD11 deficiency, severe eczema, dupilumab, HPV driven carcinoma, anal carcinoma, mycosis fungoides, hyper-IgE-syndrome, CADINS

## Abstract

**Background:**

Atopic dermatitis (AD) affects up to 25% of children and 10% of adults in Western countries. When severe or recurrent infections and exceedingly elevated serum IgE levels occur in AD patients, an inborn error of immunity (IEI) may be suspected. The International Union of Immunological Societies classification lists variants in different genes responsible for so-called Hyper-IgE syndromes. Diagnosing an underlying IEI may influence treatment strategies.

**Methods:**

Clinical and diagnostic workup of family members are presented including a detailed immunological description and histology of the carcinoma. Functional testing of the novel variant in *CARD11* underlying ‘CARD11-associated atopy with dominant interference of NF-kB signaling’ (CADINS) was performed.

**Results:**

We report on an 18-year-old patient with a long-standing history of infections, accompanied by hypogammaglobulinemia, intermittent agranulocytosis, atopy, eosinophilia and colitis. The working diagnosis of common variable immunodeficiency was revised when a novel heterozygous *CARD11* variant [c.223C>T; p.(Arg75Trp)] was identified. Functional studies confirmed this variant to have a dominant negative (DN) effect, as previously described in patients with CADINS. Five other family members were affected by severe atopy associated with the above variant, but not hypogammaglobulinemia. Malignancies occurred in two generations: an HPV-positive squamous cell carcinoma and a cutaneous T-cell lymphoma. So far, one patient is under treatment with dupilumab, which has shown marked benefit in controlling severe eczema.

**Conclusion:**

The phenotypic spectrum associated with heterozygous *CARD11* DN mutations is broad. Partial T-cell deficiency, diminished IFN-γ cytokine and increased IL-4 production, were identified as disease-causing mechanisms. Malignant disease associated with germline *CARD11* DN variants has only been reported sporadically. HPV vaccination in teenage years, and cytology screening analogous with routine cervical swabs may be recommended. Treatment with dupilumab, a monoclonal antibody blocking interleukin-4- and interleukin-13 signaling, may be of benefit in controlling severe and extended AD for some patients as reported for STAT3 loss-of-function.

## Introduction

Atopic dermatitis (AD) is one of the most common childhood diagnoses in Western European countries. It affects up to 25% of children and 10% of adults (2). AD is a common type of eczema; other atopic diseases (e.g. asthma, rhinitis, food allergies) are often associated. Certain inborn errors of immunity (IEI) may also present with eczema and highly elevated serum IgE levels. However, patients with classic IEI such as severe combined immunodeficiency (SCID) or with Omenn, Wiskott-Aldrich, DiGeorge syndromes or DOCK8 deficiency usually manifest in early infancy or childhood, accompanied by infection susceptibility in addition to early-onset severe AD. Other so-called Hyper IgE syndromes (HIES) include susceptibility to bacterial, fungal or viral infections such as molluscum contagiosum and the human papilloma virus (HPV). These include classical autosomal dominant HIES due to *STAT3* loss-of-function (LOF) and autosomal recessive entities linked to variants in *ZNF341*, *PGM3*, and genes more recently associated with HIES such as *SOCS1* genes and *CARD11* (Caspase recruitment domain family member 11) (3–7). Autosomal dominant *CARD11* variants were first published in 2017 in children and teenagers with the triad of AD/asthma, Hyper-IgE and recurrent infections (8).

CARD11, a multi-domain scaffold protein, belongs to the membrane-associated guanylate kinase (MAGUK) protein family as well as the caspase-associated recruitment domain (CARD) protein family. The protein structure is illustrated in **Supplementary Figure 1**. As a constituent of the CARD11-BCL10-MALT1 (CBM) complex, it plays a critical role in intracellular signaling cascades, most notably N**F-**κB activation downstream of antigen receptor signalling in lymphocytes. The phosphorylation of CARD11 leads to a conformational change which allows CARD11 to recruit cofactors including BCL10, MALT1 and TRAF6. This multi-protein signalosome then activates the IKK complex, leading to phosphorylation of the inhibitory IκBα protein, its ubiquitinylation, and subsequent proteasomal degradation. Activated NF-κB translocates to the nucleus and regulates transcription of proliferative, pro-inflammatory, and anti-apoptotic genes essential for lymphocyte function. The CBM complex is also involved in the activation of c-Jun N-terminal kinase (JNK) and mechanistic target of rapamycin (mTORC1) signalling following T cell receptor (TCR) engagement (9).

The phenotypic spectrum for patients with germline variants in *CARD11* is broad. Bi-allelic loss-of-function variants cause a form of severe combined immunodeficiency (SCID, MIM: 615206) (10), whereas heterozygous gain-of-function (GOF) variants result in a B-cell lymphoproliferative disease known as BENTA (B-cell expansion with NF-κB and T-cell anergy, MIM: 616452) (11). More recently, heterozygous LOF, dominant negative (DN) variants were shown to drive an autosomal dominant HIES featuring atopy and hypogammaglobulinemia (MIM: 617638) (8). Individuals with CARD11-associated atopy with dominant interference of NF-κB signaling (CADINS) disease often appear with AD, but signs and symptoms may vary between kindreds and even within families, encompassing atopic disease such as asthma or allergies, autoimmune diseases, and infections such as viral skin and respiratory tract infections. These clinical manifestations may be associated with immunologic abnormalities, such as hypogammaglobulinemia, neutropenia or abnormal T-cell proliferation and differentiation (1, 12).

## Results

### Patient Clinical and Immunological Characteristics

**Figure 1:**
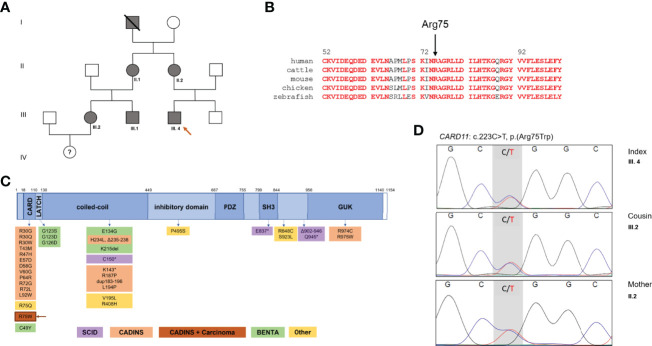
Family pedigree and CARD11 domains in health and diseases. **(A)** Family pedigree. **(B)** Multiple species alignment of CARD11 protein (AA 52-101) showing high conservation of Arg75 across species. Red: full conservation. **(C)** Schematic representation of CARD11, with functional domains and reported variants, including monoallelic gain-of-function (GOF) variants in BENTA, biallelic LOF variants in SCID, monoallelic DN LOF variants in CARD11-associated atopy with dominant interference of NF-κB signaling (CADINS), additionally monoallelic DN LOF variants in CADINS and carcinoma. DN, dominant negative; LOF, loss of function; SCID, severe combined immunodeficiency, * truncating variant. **(D)** Sanger DNA sequencing electropherograms showing the novel heterozygote variant of the *CARD11* gene in III.4, his mother II.2 and his cousin III.2.

Here we describe a novel *CARD11* variant (p.Arg75Trp) segregating with Hyper-IgE associated atopy, infections and malignancy in a three-generation kindred with at least 6 affected family members (**Figures 1A, B**).

**Figure 2:**
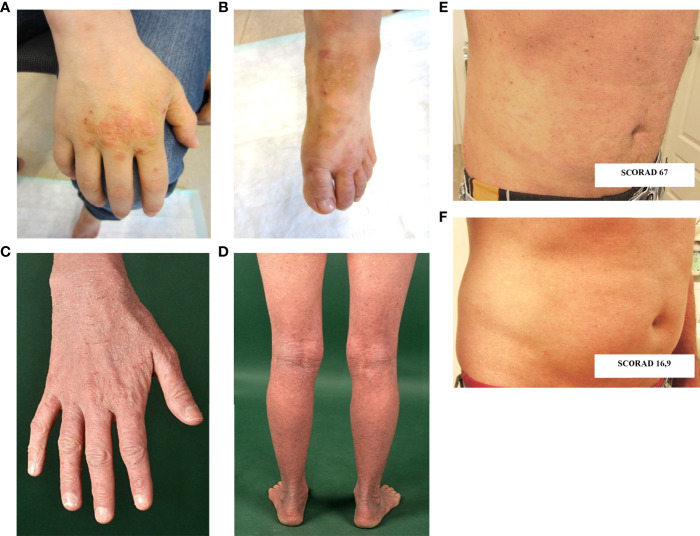
Cutaneous findings. **(A, B)** Hand and foot eczema in patient III.4. **(C, D)** Extensive eczema in patient III.2. **(E, F)** Skin findings prior **(E)** and following **(F)** treatment with dupilumab in patient III.1. SCORAD: SCORing Atopic Dermatitis; Clinical scoring system to evaluate severity of atopic eczema. Interpretation: < 25 mild, 25-60 moderate, 61-103 severe atopic eczema.

The now 18-year-old-index patient (III.4) initially presented at 2 months of age with mastoiditis, pneumonia and agranulocytosis. It was supposed to be caused by allo- or autoantibodies as neutrophil counts normalized after one year. At 2 years of age, he showed a rectal herpesvirus infection. Beginning at age 3, he suffered from recurrent respiratory infections and bronchial obstruction, and was therefore diagnosed with asthma. Severe eczema developed on his hands and feet (**Figures 2A, B**), as well as recurrent nail mycoses. He was additionally diagnosed with chronic polyposis, sinusitis and food allergies. From 12 years of age onwards, the patient complained of gastrointestinal symptoms, such as abdominal pain and nausea associated with weight loss.

At age 5, hypogammaglobulinemia was diagnosed, and the patient was started on regular subcutaneous immunoglobulin substitution. Although his susceptibility to infection markedly decreased, bronchiectasis was documented at age 13 years. Following several episodes of mild hematochezia, eosinophilic colitis was detected by endoscopy at age 16.

The maternal grandfather (I.1) had suffered from pronounced AD and Mycosis Fungoides (MF) with extracutaneous dissemination, and died at age 56.

**Figure 3:**
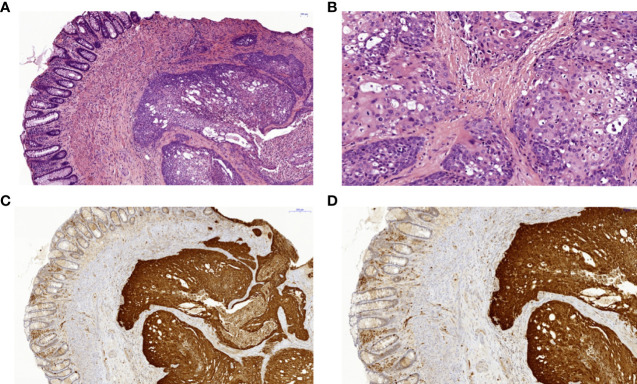
Histopathology and immunohistochemistry of a squamous cell carcinoma of the anal canal in patient II.2. **(A)** Colorectal biopsy in H&E staining: left side colorectal mucosa, right side squamous cell carcinoma. **(B)** Anal squamous cell carcinoma in H&E staining with higher magnification with cellular atypia and dyskeratoses. **(C)** Anal squamous cell carcinoma showing positive p16 immunohistochemical staining of the tumor cells as a surrogate marker for HPV-infection in 7x magnification and **(D)** in 10.6x magnification. HPV, human papilloma virus.

The index patient’s mother (II.2) suffered from severe hand and foot eczema, and recurrent respiratory infections. At age 45, she was diagnosed with anal squamous cell carcinoma induced by a local HPV18 infection (**Figure 3**). This carcinoma was discovered only after it had already metastasized, and despite removal of all intra-pelvic organs, three relapses occurred within 5 years. Clinical management was switched to palliative care.

The index patient’s maternal aunt (II.1) suffered from warts as well as from hand and foot eczema, allergies and asthma from her teenage years. Her son (III.1) and daughter (III.2) – the index patient’s cousins - report various food allergies, bronchial asthma and severe AD (**Figures 2C, D**). One of them (III.1) was successfully treated with the humanized anti-IL4Rα biological drug dupilumab, which blocks both IL-4 and IL-13 signaling. Photodocumentation pre- and post-treatment is presented in **Figures 2E, F**. The cousin’s (III.2) daughter, born in 2020, has early-onset AD and was recently found to have severe neutropenia.

In the index patient, laboratory findings were comprised of eosinophilia (up to 5900/µL [0-460]), elevated serum IgE up to max 1970 kU/L [<20] and decreased levels of IgG and IgM (**Table 1**).

**Table 1:** Summary of clinical and immunological characteristics.

	Index patient III.4	Patient's mother^2^ II.2	Patient's aunt II.1	Patient's cousin f. III.2	Patient's cousin m. III.1	Patient's grandfather I.1
**Patient characteristics**						
**Age of onset**	2 y	early childhood	14 y	early childhood	early childhood	early childhood
**Bacterial infections**	Otitis media, sinusitis	n	n	rhinoconjunctivitis	n	NA
**Viral infections**	HSV rectally	HPV (genital)	Herpes labialis	n	n	NA
**Bronchiectases**	**y**	n	n	n	n	NA
**Asthma bronchiale**	**y**	n	**y**	**y**	**y**	**y**
**Atopic dermatitis**	**y**	**y**	**y**	**y**	**y**	**y**
**Food and pollen allergies**	**y**	n	**y**	**y**	**y**	**y**
**Malignancies**	n	Anal squamous cell carcinoma	n	n	n	Mycosis fungosis, suspected T-cell lymphoma with lung metastases
**Treatment**	IgG substitution	prednisolone, chemotherapy, radiation	prednisolone, antihistamines	ciclosporine A (stopped after 2 years), budesonide, topic prednisolone, antihistamines	salbutamol, prednisolone, topical tacrolimus, **dupilumab**	no records available
**Alive**	y	n (deceased at age 50)	y	y	y	n (deceased at age 56)
**Descriptive and functional parameters**						
***Blood count***						
**Total leukocytes (cells/µL)**	6600 [4400-8100]	5000 [4400-11300]	5700 [4400-11300]	**12000** [4400-11300]	9970 [4400-11300]	NA
**Eosinophils (cells/µL)**	**5900** [0-650]	130 [0-490]	38 [0-490]	100 [0-490]	227 [0-490]	NA
**Neutrophils (cells/µL)**	494 [165-798]	342 [180-755]	275 [180-755]	**778** [180-755]	521 [180-755]	NA
**Lymphocytes (cells/µL)**	1980 [1400-3300]	**750** [1400-2800]	2052 [1400-2800]	2040 [1400-2800]	1904 [1400-2900]	NA
***Lymphocyte subsets***						
**CD3+ (cells/µL)**	1663 [1000-2200]	**495** [1000-1980]	1847 [1000-1980]	1714 [1000-1980]	1607 [1000-1980]	NA
**CD4+ (cells/µL)**	1228 [530-1300]	**360** [630-1120]	**1703** [630-1120]	**1346** [630-1120]	1325 [630-1120]	NA
**CD8+ (cells/µL)**	396 [330-920]	**128** [240-700]	**115** [240-700]	367 [240-700]	263 [ 240-700]	NA
**CD4/CD8 ratio**	**3,1** [1-2,5]	2,8 [1,3-3,5]	**14,82** [1,3-3,5]	3,667 [1,3-3,5]	5 [1,3-3,5]	NA
**CD45 RA+ (% CD4+)**	60 [33-66]	21 [23-69]	**73** [12-69]	70 [23-69]	77,2 [2-68]	NA
**CD45 RO+ (% CD4+)**	31 [26-63]	71 [30-74]	**21** [30-74]	**22** [30-74]	22,5 [30-74]	NA
**CD3+/DR+ in %**	**11,7** [0,3-3,7]	**22** [0-7%]	**7,3** [0-7%]	6,5 [0-7%]	11,9 [0-7%]	NA
**NK cells (CD3-CD16+CD56+ cells/µL)**	**75** [90-700]	**51** [80-630]	**14** [80-630]	98 [10-130]	196 [80-630]	NA
**B cells (CD19+ cells/µL)**	238 [110-570]	188 [90-320]	121 [90-320]	147 [90-320]	105 [90-320]	NA
**transitional B cells (IgM++/CD38++ of %CD19+)**	4,9 [0,9-5,7]	3,6 [0,9-5,7]	1,8 [0,9-5,7]	**0,8** [0,9-5,7]	4,9 [0,9-5,7]	NA
**naive B cells (IgD+CD27+ of %CD19+)**	**92** [52-87]	80 [52-87]	85 [52-87]	**90** [52-87]	72 [33-100]	NA
**class-switched (IgM-/CD20+/CD27+ of %CD19+)**	**2,7** [3,8-23]	4,7 [3,8-23]	7,7 [3,8-23]	5 [3,8-23]	14,1 [3-46]	NA
***Intracellular cytokine measurement***						
**Th1 (% of IFN_γ_ expression in CD3+)**	40,9 [30-51]	NA	40,5 [30-51]	**24,4** [30-51]	NA	NA
**Th2 (% of IL-4 expression in CD3+)**	0,75 [0,12-0,78]	NA	**1,91** [0,12-0,78]	**3,21** [1,12-0,78]	NA	NA
**Th17 (% of IL-17 expression in CD3+)**	3,12 [1,41-4,73]	NA	1,94 [1,41-4,73]	4,72 [1,41-4,73]	NA	NA
***T-cell proliferation***						
**IL-2**	↓	↓	↓	normal	NA	NA
**PHA**	normal	normal	normal	normal	normal	NA
**CD3/CD28 beads**	normal	normal	normal	normal	normal	NA
**MLC pool**	↓	↓	↓	↓	NA	
**Tetanus antigen**	↓	↓	↓	↓	↓	NA
**CMV antigen**	↓	↓	↓	↓	↓	NA
**Adenovirus antigen**	normal	normal	↓	↓	↓	NA
**Candidin antigen**	↓	↓	↓	↓	↓	NA
***Immunoglobulins***						
**IgG (g/L)**	7,1¹ [7-16]	6,9 [7-16]	8,43 [7-16]	8,64 [7-16]	11,89 [7-16]	NA
**IgM (g/L)**	<0,16 [0,4-2,3]	<0,06 [0,4-2,3]	0,7 [0,4-2,3]	0,37 [0,4-2,3]	0,72 [0,4-2,3]	NA
**IgE (kU/L)**	1970 [<20]	<2 [<20]	1973 [<20]	4798 [<20]	575 [<20]	NA
**IgA (g/L)**	2,07 [0,7-4]	1,48 [0,7-4]	2,22 [0,7-4]	4,36 [0,7-4]	6,39 [0,7-4]	NA

¹Under immunoglobulin substitution; ²Sample taken after radiotherapy; normal >30% of healthy control; MLC, mixed lymphocyte culture, NA, not available; y, yes; n, no; ↓ - reduced; bold values – abnormal results.

A recent immunologic workup of the index patient revealed reduced NK cells, but normal T- and B-cell counts. Elevated levels of activated CD3+ T-cells, predominantly HLA-DR+CD8+ T-cells were observed, whereas the proportion of naïve T cells was normal. The proportion of naive B-cells was elevated, but class-switched memory cells were significantly reduced (**Table 1**). Hypogammaglobulinaemia and poor B-cell maturation were detected in the index patient only.

Other family members (II.1 and 2, III.1 and 2) also show increased IgE levels, activated HLA-DR+CD3+ T cells, effector memory CD45RA re-expressing CD8+ T-cells (TEMRA) and an elevated CD4/CD8 ratio, but markedly reduced T-cell function: decreased proliferation upon stimulation with IL-2, in a mixed lymphocyte culture and to specific antigens. Intracellular cytokine measurements indicated elevated IL-4 production in 2/3 patients tested, suggesting skewed Th2 differentiation (**Table 1**).

Whole-exome sequencing (WES) revealed a heterozygous variant in *CARD11* (NM_032415.7: c.223C>T; p.Arg75Trp). This variant is absent from the gnomAD database, and is predicted to be damaging based on several *in-silico* algorithms (Polyphen2, SIFT MutationTaster), including a high CADD score of 29 (v1.6). The variant was also confirmed by Sanger sequencing in the mother, aunt and cousins of the index patient (**Figure 1D**). No other suspicious genetic variants were found by WES. The variant was submitted to the ClinVar database (SUB11100602).

**Figure 4:**
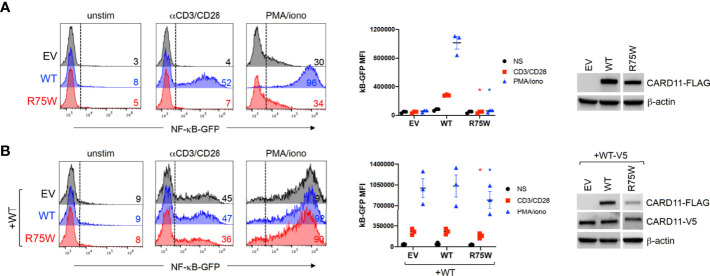
Functional testing of R75W CARD11. JPM50.6 cells were transfected with EV, WT or R75W CARD11-FLAG expression constructs, without **(A)** or with **(B)** a WT CARD11-V5 construct. After 24 hrs, transfectants were stimulated with anti-CD3/CD28 Abs or PMA/ionomycin for 24 hrs, and GFP expression was measured by flow cytometry. Histograms from one representative experiment (left panels) denote %GFP+ cells -/+ stimulation. Mean fluorescence intensity (MFI) of GFP+ cells was graphed for 3 independent experiments (middle panels); asterisks denote statistically significant decreases in GFP MFI for R75W relative to WT -/+ WT CARD11-V5 (paired t-test, *p <*0.04). CARD11 protein expression was measured in cell lysates by immunoblotting; β-actin serves as a loading control.

Arg75 is a highly conserved amino acid residue located in the CARD domain, a region that is depleted of missense variation (**Figure 1C** and **Supplementary Figure 2**). Indeed, a recent multiplexed assay of variant function identified Arg75 as one of many hotspots for LOF variants in the CARD domain of *CARD11* (13), and a separate amino acid exchange at this codon (p.Arg75Gln) was previously confirmed as DN in a patient with neutropenia (11). To formally test the impact of this novel variant on *CARD11* signaling, we transfected JPM50.6 cells with expression plasmids encoding WT and/or R75W CARD11 and measured NF-κB driven GFP expression following T-cell activation (3). Relative to WT, no appreciable NF-κB induction was noted with R75W CARD11 (**Figure 4A**). Moreover, R75W demonstrated mild DN activity in suppressing NF-κB activation when co-expressed with WT CARD11 (**Figure 4B**), with comparable expression of both proteins noted. These results confirmed R75W as a causative DN *CARD11* variant in this family.

## Discussion

This multi-generation family has clinical and immunological features in common with formerly described patients suffering from CADINS disease (1, 8). As described for other *CARD11* DN variants, there is a high penetrance but variable intrafamilial expressivity caused by the (p.Arg75Trp) variant. As an infant, the index patient had atopic dermatitis-like rash, but was more severely affected by infections and early-onset asthma. Other family members suffered from moderate to severe atopic symptoms that raised no suspicion for an IEI. The agranulocytosis of the index patient was detected at three months of age, after one year the neutrophil count had normalized. Dorjbal et al. observed neutropenia in 4 patients with functional DN variants not associated with bone marrow abnormalities suggesting an autoimmune origin (1).

Although T-cell subpopulations were normal in numbers and distribution, the proliferation of the index patient’s T cells was impaired upon stimulation with mitogens such as IL-2 and in mixed lymphocyte culture, but also after stimulation with specific antigens such as tetanus, CMV and candidin. This was also observed in all other affected family members and is consistent with impaired CARD11-dependent T-cell activation after TCR (CD3) and CD28 ligation. Indeed, impaired T-cell function predisposes to viral infections, and poor CD28 stimulation seems to have a special significance in the predisposition for HPV infection (14, 15). Apart from CARD11 deficiency, signaling *via* CD28 is also disturbed in other IEI such as CD28, MAGT1 and CARMIL2 deficiencies, and affected patients have been documented with HPV-driven warts (1, 16, 17). In a cohort of 44 individuals with CADINS disease, cutaneous infections were the second most common manifestation (68%) after AD (73%). Viral skin infections included common HPV-driven warts in over 25% of patients with *CARD11* DN variants (1). HPV is known to cause malignancies like cervical cancer and is linked to more than 90% of all anal carcinomas (18) e.g. in HIV positive individuals. These findings highlight a possible relationship between this germline *CARD11* variant and the HPV18 positive anal carcinoma in the index patient’s mother. HPV16 has been identified as the predominant HPV type in anal squamous cell carcinoma, followed to a lesser extent by HPV18, HPV33, HPV31, HPV6, HPV58 and HPV35 (19). HPV vaccination which is 9-valent (HPV types 6, 11, 16, 18, 31, 33, 45, 52, and 58) may offer an option for primary prevention of HPV induced carcinomas in patients with CADINS in analogy to HIV patients. Likewise, routine screening including anoscopy and anal cytology may be warranted in CADINS patients (16, 18).

Somatic *CARD11* variants have been associated with several lymphoid malignancies (20). Somatic GOF *CARD11* mutations were initially described in ~10% of diffuse large B-cell lymphomas associated with constitutive NF-κB activity (21). Moreover, hypermorphic/GOF variants in *CARD11* and other non-canonical NF-κB genes were also reported in patients with MF, Sézary Syndrome, and HTLV-1-driven adult T-cell lymphoma (20, 22, 23). More recently, somatic variants in *CARD11* were found to be associated with solid tumors such as human sinonasal tumors (24). Watt *et al.* showed that in cutaneous squamous cell carcinomas, CARD11 protein expression is increased, and novel *CARD11* variants outside the coiled-coil domain led to constitutively activated NF-κB signaling (25). By contrast, germline *CARD11* LOF/DN variants were previously discovered in 2 patients with peripheral T-cell lymphoma (p.Glu57Asp) and MF (p.Arg187Trp) (11). Our findings further underscore a perplexing connection between *attenuated* CBM signaling and malignancy, perhaps linked to infectious triggers (e.g. HSV-1) and/or impaired T-cell surveillance in the skin. More work is required to decipher how defects in distinct CARD11-dependent signaling pathways may explain this surprising phenomenon.

Dupilumab, a humanized monoclonal antibody against IL-4Rα, is increasingly being used in treatment-refractory AD, including in children from the age of 6 years. The imbalance towards Th2 cytokine production in *CARD11* DN patients suggests that this biological may be useful in controlling AD in IEI with atopy. Recently, it has been successfully administered in the treatment of AD in monogenic forms of HIES, such as STAT3 LOF (3) and also in one individual with a DN *CARD11* variant (26). Moreover, a recent meeting report described three patients with CADINS disease and severe AD ameliorated by treatment with dupilumab or omalizumab (27).

In summary, we describe a multi-generation family with CADINS disease caused by a novel *CARD11* DN variant. The clinical phenotype is not only characterized by severe atopy (AD, asthma, food allergies) and recurrent infections in the context of both hypogammaglobulinemia and reduced T-cell responses to specific antigens, but also through the occurrence of 2 different malignancies: an HPV-driven anal squamous cell carcinoma and skin infiltrating T-cell lymphoma in one family member each. Dupilumab is likely helpful in alleviating AD in some CADINS patients.

## Methods

Patients were enrolled on protocols approved by ethics review committees and thereupon provided written informed consent.

### Immunophenotyping

Patient peripheral blood mononuclear cells (PBMC) were isolated by density-gradient centrifugation using Biocell separating solution (density 1.077 g/ml, Biocell) and resuspended. Surface immunophenotyping was performed in the following manner: for T-cell panel - 100µl blood (EDTA) were used, applying BD FACS Lyse Wash Assistant, for B-cell panel - BD Vacutainer CPT 4mL was used, preparation of peripheral blood mononuclear cells (PBMCs) was performed according to manufacturer’s protocol. Antibodies used: CD45, CD14, CD19, CD20, CD27, CD16/56, CD3, CD4, CD8, TCRab, TCRgd, HLA-DR, CD45RA, CD45RO, CCR7, CD31, CD21, CD24, CD38, IgD, IgG, IgM (Becton Dickinson GmbH, Heidelberg, Germany), IgA (BIOZOL Diagnostica Vertrieb GmbH, Eching, Germany). Samples were acquired and analysed with BD FACSLyric Flow Cytometer using the BD FACSuite V1.4.0 Analysis-Software.

### Intracellular Cytokine Staining

Intracellular cytokine measurement was performed according to Körholz et al., 2021 (7). Briefly PBMCs (1x10^6^/ml) were left unstimulated or stimulated with 10 ng/ml PMA (Phorbol-12-Myristat13-Acetat) and 1µg/ml Calcium-Ionophore (both Merck KGaA, Darmstadt, Germany) under addition Brefeldin A (1µl/ml, BD-Biosciences San José, CA) for 12h overnight. Cells then were harvested and washed twice with PBS/1%FCS. For surface staining, cells were incubated with anti-CD45RO-PE (5µl/test) (Biolegend, San Diego, CA), anti-CD3-APC (2µl/test), anti-CD4-APC-AlexaFluor750 (5µl/test) and anti-CD45-Krome Orange (5µl/test) (Beckman Coulter, Krefeld, Germany) for 30 min at 4°C. After beeing washed twice with PBS/1% FCS, cells were fixed and permeabilized with 100µl Cytofix/Cytoperm™ (BD Biosciences, San José, CA) for 20 min at 4°C and then washed twice according to the manufacturers instructions. For intracellular staining, cells were incubated with anti-IFNγ-FITC (5µl/test), anti-IL4-PE Cy7 (5µl/test) (both Biolegend) and anti-IL-17A eFlour 450 (5µl/test) (eBioscience/Thermo Scientific) and anti-CD4 APC-AlexaFluor750 (2µl/test) (Beckman-Coulter, Krefeld,Germany) for 45min at 4°C. Cells were washed twice with Perm/Wash Buffer (BD Biosciences) and diluted in 500µl PBS/1% FCS. Analysis was performed on a BD LSR II.

### T-Cell Proliferation

T-cell functions were assessed using a standard 3-H-Thymidin proliferation assay: 10^5^ cells/well were stimulated in triplicates in a 96 well flat bottom plate with IL2 (100iE/well, Pepro Tech, New Jersey, US), PHA (1,5%, Invitrogen, Carlsbad, CA, USA), CD3/28 beads (12,5µl/ml, Thermo Scientific/Gibco Waltham, MA, USA), Tetanus antigen (50µl/ml, Statens Serum Institut, Kopenhagen, Denmark), CMV antigen, Adenovirus antigen (both: Institute for Virology, Ulm University). As a positive control, buffy coat PBMC of healthy donors were stimulated. After 3 days of incubation for unspecific stimulation (IL2, PHA, CD3/28) and 5 days for antigen-specific stimulation (Tetanus, viral antigens) at 37°C/5% CO_2_, ^3^H-thymidin (0,001mCi/ml, Perkin Elmer Germany) was added for 18h. Cells were harvested and incorporated ^3^H-thymidin was measured in a beta counter (Microplatecounter TopCount NCT, Packard/Perkin Elmer). Stimulation indices were calculated as cpm mean (of triplicates) after stimulation/cpm mean of negative controls (culture-medium alone).

### Molecular Genetics Analysis

Genomic DNA from the index patient was isolated from peripheral blood and coding genes enriched using the SureSelect Human All Exon Kit V6 (Agilent technologies) for subsequent WES on the Illumina system. Reads were aligned to the human genome build GRCh37/hg19. Sequence reads were called and analyzed according to an in-house standard operating procedure using the VarFish platform (28). Variants were filtered by minor allele frequency, mode of inheritance, functional prediction and constraint metrics, such as LOEUF score and pLI score for LoF-variants and Z-Score for Missense variants (29). Confirmation of the *CARD11* variant and segregation analysis was done by Sanger sequencing using the following primers for amplification and sequencing: Ex4.F:5´-GACTTGCGTTCCATCAGATATGT-3´, Ex4.1R:5´- ACACGCCCCTCCTCTTAGAG-3´.

### Cell Transfections

pUNO-CARD11-FLAG plasmids encoding the R75W CARD11 protein were produced by site-directed mutagenesis as previously described (11). JPM50.6 T cells (3 million) were transfected with 3 μg empty pUNO vector (EV), WT or R75W CARD11 plasmids, with or without WT CARD11-V5 using an ECM 630 electroporator (Harvard BTX, Holliston, MA, USA) at 260V, 950 μF (11). At 24 hrs post-transfection, cells were stimulated for 24 hrs with 1 mg/ml of anti-CD3 (clone HIT3a) + anti-CD28 (clone CD28.2) antibodies (BD Biosciences), 20 ng/ml phorbol myristate acetate (PMA) + 1 mM ionomycin (Millipore Sigma, St. Louis, MO, USA) or left unstimulated. NF-κB-dependent GFP reporter expression was measured using an Accuri C6 flow cytometer (Becton Dickinson). CARD11 protein expression was assessed in transfectant lysates by immunoblotting as previously described (11).

### Histology and Molecular Pathology

The index patient’s mother had a recurrent moderately well differentiated squamous cell carcinoma of the anal canal which was operated and routinely processed. The postoperative tumor classification was: rpT4, rpNx, rL0, rV0, rPn0, Rx, G2. **Figure 3** shows a different magnification of the H&E sections and a strong and diffusely positive immunohistochemical reaction for p16 (Cytomed mouse monoclonal antibody clone JC2; 1:100, OPTIview detection system on a VENTANA platform) which was used as a surrogate marker for HPV-infection.

DNA was extracted from the tumor sample for HPV-PCR and subsequent typing by means of the HPV 3.5 LCD-array Kit (Chipron). Typing revealed infection with the carcinogenic HPV-subtype HPV18.

## Conclusion

Patients with CARD11-associated atopy and dominant interference of NF-κB signaling (CADINS) disease, were first described in 2017. They usually present with varying degrees of atopy and cutaneous viral and respiratory infections with or without hypogammaglobulinemia. Our study expands the clinical spectrum of CADINS patients by reporting a multi-generation family with Hyper-IgE, disturbed T-cell function in response to IL-2, MLC and specific antigens, hypogammaglobulinemia and malignancies. Poor CD28 signaling may explain predisposition to HPV infection. An HPV triggered squamous cell carcinoma has, however, not been reported in CARD11 deficiency before. This finding underscores a perplexing connection between attenuated CBM signaling and malignancy, perhaps linked to infectious triggers and/or impaired T-cell surveillance. Further investigation is warranted to elucidate how defects in distinct CARD11-dependent signaling pathways may explain this surprising phenomenon. Dupilumab is likely helpful in alleviating severe AD in some CADINS patients.

## Data Availability Statement

The datasets for this article are not publicly available due to concerns regarding participant/patient anonymity. Requests to access the datasets should be directed to the corresponding author.

## Ethics Statement

The studies involving human participants were reviewed and approved by Ethikkommission an der TU Dresden. The patients/participants provided their written informed consent to participate in this study. Written informed consent was obtained from the individual(s) for the publication of any potentially identifiable images or data included in this article.

## Author Contributions

LP and JK, conceptualization, data collection, writing, and editing. FB, formal analysis (interpretation of exome data), software, and visualization. MS, methodology and HPV sequencing. DP, technical assistance and methodology. EK, patient care, methodology, and photodocumentation. CK, ML, and RB, patient care and editing. EJ, data curation and investigation. JR, methodology, writing, editing, and treatment of the index patient. BD, DY, and AS, investigation, functional testing of the variant, and editing. DA, methodology, data interpretation, and editing. VG and ML-K, Sanger sequencing, editing, and funding acquisition. CS, conceptualization, data curation, writing-editing, supervision, and funding acquisition. All authors contributed to the article and approved the submitted version.

## Funding

This work was supported by a grant from the Deutsche Forschungsgemeinschaft (CRC237 369799452/B21) to ML-K, by the Rosemarie-Germscheid Stiftung to CS, by a grant from the Else-Kröner Fresenius clinician-scientist program to JK, and a Specific Defect Research Program grant from the Jeffrey Modell Foundation to AS.

## Conflict of Interest

The authors declare that the research was conducted in the absence of any commercial or financial relationships that could be construed as a potential conflict of interest.

## Publisher’s Note

All claims expressed in this article are solely those of the authors and do not necessarily represent those of their affiliated organizations, or those of the publisher, the editors and the reviewers. Any product that may be evaluated in this article, or claim that may be made by its manufacturer, is not guaranteed or endorsed by the publisher.

## Supplementary Material

Supplementary Figure 1 Structural modeling of the CARD11 protein by AlphaFold (https://alphafold.ebi.ac.uk/entry/Q9BXL7). Positively charged amino acid Arg75 forms hydrogen bonds with negatively charged amino acid Glu59, likely important for protein stability of the CARD domain.

Supplementary Figure 2 Screenshot from DECIPHER - https://www.deciphergenomics.org/gene/CARD11/overview/protein-genomic-info - showing domain structure, regional missense constraint scores and ClinVar annotated pathogenic variants for the *CARD11* gene. Notably, amino acid residues 1-140 (including the CARD domain) are depleted of missense variation (calculated “MPC”of 0.1 indicated by black asterisk – data retrieved from ExAC). Pathogenic missense variants cluster within the CARD domain. Black triangle points towards the location of the variant described in this study (p.Arg75Trp).
