# Supplementary figures and images for: Hyper-IgE and Carcinoma in CADINS Disease
Source: Front Immunol. 2022 May 16;13:878989. doi: 10.3389/fimmu.2022.878989 (PMC9149281; doi:10.3389/fimmu.2022.878989)

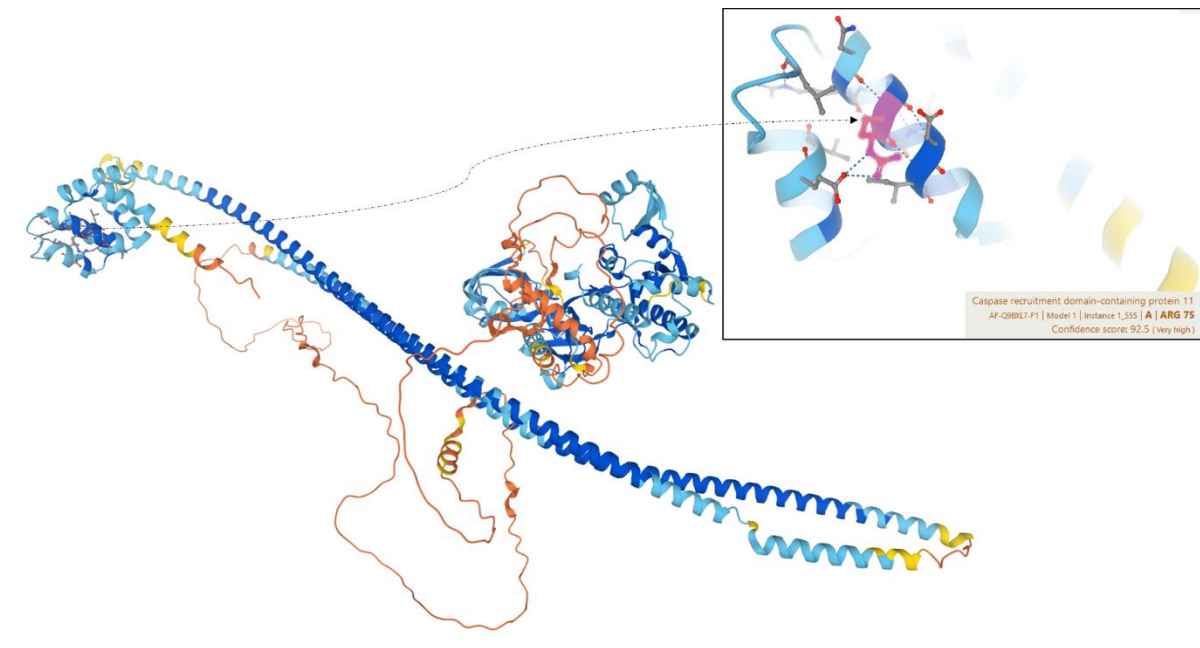

Suppl. Figure 1

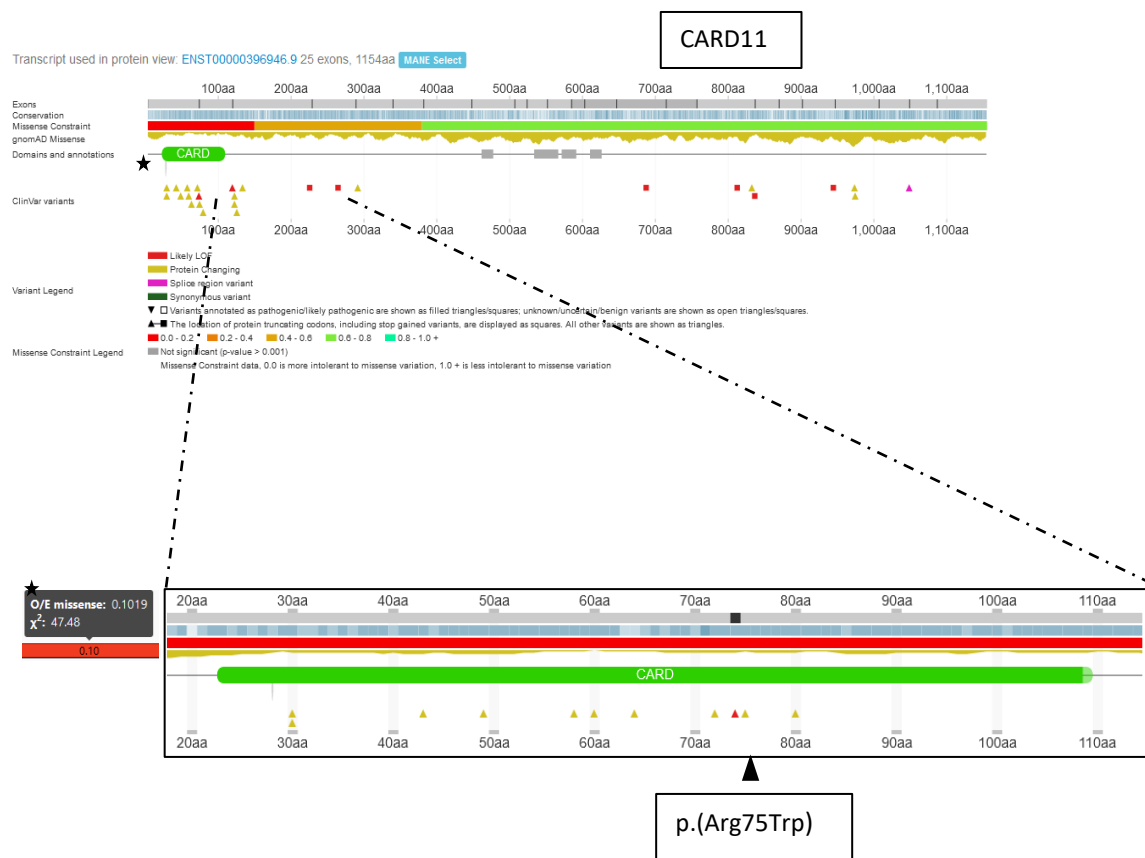

Suppl. Figure 2

Supplement: Supplementary Figure 1 — Structural modeling of the CARD11 protein by AlphaFold (https://alphafold.ebi.ac.uk/entry/Q9BXL7). Positively charged amino acid Arg75 forms hydrogen bonds with negatively charged amino acid Glu59, likely important for protein stability of the CARD domain. [file DataSheet_1.pdf]
